# Supplementary material for: Adaptation of a school-based mental health program for adolescents in Vietnam
Source: PLoS One. 2022 Aug 4;17(8):e0271959. doi: 10.1371/journal.pone.0271959 (PMC9352022; doi:10.1371/journal.pone.0271959)
Supplement: Attachment — Submitted filename: Responses to reviewers_6_June.docx [file pone.0271959.s001.docx]

6/2/2022

Professor Bronwyn Myers
Academic Editor
PLOS ONE

Dear Professor Myers,

Re: PONE-D-22-01625 A school-based mental health program for adolescents in Vietnam: adaptation, relevance, comprehensibility and acceptability

We would like to thank you and the reviewers for the constructive comments and suggestions. The feedback is really helpful in improving our manuscript. We have carefully amended the manuscript accordingly (in tracked changes) and hope that it is now suitable for publication in PLOS One. Our response to each comment is detailed below.

Yours sincerely,

(On behalf of the authors)

Nga La

**Journal Requirements:**

*1. Please amend your current ethics statement to address the following concerns:
a) Did participants provide their written or verbal informed consent to participate in this study?
b) If consent was verbal, please explain i) why written consent was not obtained, ii) how you documented participant consent, and iii) whether the ethics committees/IRB approved this consent procedure.*

Response: We have added a statement in the manuscript. It reads “Every participant provided written informed consent to participate in this study”. (Page 12)

2. *We note that your paper includes detailed descriptions of individual patients/participants. As per the PLOS ONE policy (*[*http://journals.plos.org/plosone/s/submission-guidelines#loc-human-subjects-research*](http://journals.plos.org/plosone/s/submission-guidelines#loc-human-subjects-research)*) on papers that include identifying, or potentially identifying, information, the individual(s) or parent(s)/guardian(s) must be informed of the terms of the PLOS open-access (CC-BY) license and provide specific permission for publication of these details under the terms of this license. Please download the Consent Form for Publication in a PLOS Journal (*[*http://journals.plos.org/plosone/s/file?id=8ce6/plos-consent-form-english.pdf*](http://journals.plos.org/plosone/s/file?id=8ce6/plos-consent-form-english.pdf)*). The signed consent form should not be submitted with the manuscript, but should be securely filed in the individual's case notes. Please amend the methods section and ethics statement of the manuscript to explicitly state that the patient/participant has provided consent for publication: “The individual in this manuscript has given written informed consent (as outlined in PLOS consent form) to publish these case details*

Response: We have removed all data from the pilot study to publish in another paper, as the reviewers have suggested. Therefore, no detailed descriptions of individual participants are included in this paper.

3. *We note that you have stated that you will provide repository information for your data at acceptance. Should your manuscript be accepted for publication, we will hold it until you provide the relevant accession numbers or DOIs necessary to access your data. If you wish to make changes to your Data Availability statement, please describe these changes in your cover letter and we will update your Data Availability statement to reflect the information you provide*

Response: We would like to change the Data Availability statement to “The interview and group discussion transcripts are available to other researchers upon request, due to ethical restrictions on data sharing”.

*4. We note that Figure(s) 1 and 2 in your submission contain copyrighted images. All PLOS content is published under the Creative Commons Attribution License (CC BY 4.0), which means that the manuscript, images, and Supporting Information files will be freely available online, and any third party is permitted to access, download, copy, distribute, and use these materials in any way, even commercially, with proper attribution. For more information, see our copyright guidelines:* [*http://journals.plos.org/plosone/s/licenses-and-copyright*](http://journals.plos.org/plosone/s/licenses-and-copyright)*.
 
We require you to either (1) present written permission from the copyright holder to publish these figures specifically under the CC BY 4.0 license, or (2) remove the figures from your submission:
 
1. You may seek permission from the original copyright holder of Figure(s) 1 and 2 to publish the content specifically under the CC BY 4.0 license. 
 
We recommend that you contact the original copyright holder with the Content Permission Form (*[*http://journals.plos.org/plosone/s/file?id=7c09/content-permission-form.pdf*](http://journals.plos.org/plosone/s/file?id=7c09/content-permission-form.pdf)*) and the following text:
“I request permission for the open-access journal PLOS ONE to publish XXX under the Creative Commons Attribution License (CCAL) CC BY 4.0 (*[*http://creativecommons.org/licenses/by/4.0/*](http://creativecommons.org/licenses/by/4.0/)*). Please be aware that this license allows unrestricted use and distribution, even commercially, by third parties. Please reply and provide explicit written permission to publish XXX under a CC BY license and complete the attached form.”
 
Please upload the completed Content Permission Form or other proof of granted permissions as an "Other" file with your submission. 
 
In the figure caption of the copyrighted figure, please include the following text:* *“Reprinted from [ref] under a CC BY license, with permission from [name of publisher], original copyright [original copyright year].”*
 

Response: Figure 1: Process of translation and cultural adaptation of the Resourceful Adolescent Program (RAP) was developed for this manuscript. One of the authors of this manuscript is also an author of Figures 2, 3, and 4. We have attached the completed Content Permission Form for these figures. Figure captions have been added.

Reviewers' comments:

Reviewer #1: 

*Comment 1.1. Perhaps one way to approach this is a summary paragraph at the beginning of the results section that walks the reader through the various adaptions and then have sections by each of those adaptions area. Or perhaps a table of the changes (similar to Table 1) where you document the components of the RAP-A program and then your adaptions on the left under "Happy House". Something very simplistic for the table such as "Program Name; RAP'A; Happy House"; then "Group size; x; x" and "Age group; 12-15 years; 11-18"; etc. This will help the reader quickly see what changed and the differences. Then your results section can discuss in more details.*

Response: Many thanks for this suggestion. We have added a brief written summary and a table of the changes at the beginning of the results section. (Page 13)

*Comment 1.2. Lastly, I feel this is two papers in one. You should have one paper on the changes and the second on the pilot data. Right now having the two in one paper is too much and the details of the pilot get lost. Each of these (the modifications, and the pilot) deserve their own paper.

For the pilot test outcomes paper your table 1 should be the demographics of who the students/teachers were. This way others that may want to use your program know more details about the demographics of your population and then can generalize them (or not) to their community.*

Response: Thank you for this feedback. We have removed the pilot data from this manuscript and will report it in another one.

*Comment 1.3. Example of places that need flushed out more:
• Page 13, lines 256-7: Authors state "several issues arose"; however, the only problem discussed below is the program name. Can the authors please give more details regarding the "issues"?*

Response: We have added a brief summary of the issues and a table (Table 1) to clarify the issues that arose in the translation and adaptation process (Page 13).

*Comment 1.4. Page 17, line 306: "some examples". Again, please be clear what was changed.*

Response: We have added all changes into Table 4.

Reviewer #2:

*Comment 2.1* This is an interesting and important piece of work. The authors attempt to cover a lot of ground by reporting on both the adaptation process and the pilot/feasibility test of the intervention. Because of this, the paper is light on some of the details of the pilot test. I believe that this could be partitioned off to be a second paper- allowing for more detail on the pilot to be provided.

Response: We agreed with the reviewer’s suggestion and have removed the content relating to the pilot test from this paper.

*Comment 2.2. I also found the use of three approaches to cultural adaptation confusing- the rationale for using aspects of these three approaches rather than choosing one approach is not clear enough.*

Response: We have elaborated our adaptation strategies to make it clearer. We have developed a model for this study that uses principles from the three approaches (pages 8 and 9).
